# Pathologic Complete Response and Survival in Rectal Cancer: A Systematic Review and Meta-Analysis

**DOI:** 10.1001/jamanetworkopen.2025.21197

**Published:** 2025-07-16

**Authors:** Kavin Sugumar, Jessica Jin Lie, Chee-Chee Stucky, Yu-Hui Chang, Justin Brady, Nabil Wasif, Mohamad Bassam Sonbol, David Etzioni, Harvey Mamon, Tanios Bekaii-Saab, Zhi Ven Fong

**Affiliations:** 1 Department of Surgery, Tulane University, New Orleans, Louisiana; 2 Department of Surgery, University of British Columbia, Vancouver, British Columbia, Canada; 3 Department of Surgery, Mayo Arizona, Phoenix, Arizona; 4 Division of Hematology and Oncology, Department of Medicine, Mayo Clinic, Phoenix, Arizona; 5 Department of Radiation Oncology, Dana Farber Cancer Institute, Brigham and Women’s Hospital, Boston, Massachusetts; 6 Division of Surgical Oncology and Endocrine Surgery, Department of Surgery, Mayo Arizona, Phoenix, Arizona

## Abstract

**Importance:**

Pathologic complete response (pCR) is increasingly used as a surrogate end point for survival in randomized clinical trials (RCTs) in the field of gastrointestinal oncology. Although this approach has been endorsed by the US Food and Drug Administration, it is still novel and incompletely verified.

**Objective:**

To evaluate the trial-level association between pCR and survival in rectal cancer RCTs examining the effectiveness of neoadjuvant therapy.

**Data Sources:**

A meta-analysis was conducted to identify eligible RCTs in PubMed, EMBASE, and Cochrane databases published between database inception to January 3, 2024.

**Study Selection:**

RCTs evaluating neoadjuvant therapies in patients with rectal cancer who underwent subsequent surgical resection and reported pCR, overall survival (OS), and disease-free survival (DFS) were included.

**Data Extraction and Synthesis:**

Two investigators extracted the data, which were verified by a third author. The risk of bias and the certainty of the evidence were evaluated using the Cochrane Risk of Bias Tool, version 2, and the Grading of Recommendations Assessment, Development, and Evaluation tool, respectively. Adjusted odds and hazard ratios with their 95% CIs were extracted. Weighted linear regression was used to assess the correlation between pCR and both OS and DFS.

**Main Outcomes and Measures:**

Trial-level correlation between pCR and both OS and DFS.

**Results:**

Twenty-five RCTs, including 11 882 patients, met the inclusion criteria. On meta-regression analysis, pCR was not correlated with OS (β = 0.37; 95% CI, −0.98 to 1.71; *P* = .57). Similarly, pCR was not correlated with DFS (β = −0.84; 95% CI, −2.55 to 0.87; *P* = .32). Two studies (8%) used in the meta-analysis had high risk of bias. On sensitivity analysis excluding these studies, pCR was still not associated with OS or DFS. Subgroup analysis of RCTs using total neoadjuvant therapies was not possible due to limited sample size.

**Conclusions and Relevance:**

In this systematic review and meta-analysis of RCTs comparing neoadjuvant therapies in rectal cancer, there was no trial-level association between pCR and survival. These results suggest that the use of pCR as a surrogate end point for survival should be reexamined and used more cautiously.

## Key Points

**Question:**

Can pathologic complete response (pCR) be used as a surrogate end point for survival in rectal cancer?

**Findings:**

In this systematic review and meta-analysis of 25 rectal cancer trials with a total of 11 882 patients, pCR was not associated with overall survival and disease-free survival on trial-level meta-regression analysis.

**Meaning:**

This study’s findings suggest a recommendation against using pCR as a surrogate end point for survival in rectal cancer.

## Teaser

This systematic review and meta-analysis examines the trial-level association between pathologic complete response and overall and disease-free survival in neoadjuvant rectal cancer randomized clinical trials.

## Introduction

Clinical end points are used to assess the effectiveness of novel cancer therapies.^1^ Overall survival (OS) is considered the reference standard end point for estimating long-term efficacy in oncologic drug development,^2^ but measuring OS can be expensive and time-consuming and lacks information on quality of life and treatment failure.^1^ In an attempt to solve these problems, investigators have developed surrogate end points (SEPs) of long-term outcomes for various cancer types, which have been increasingly used during the past decade.^3^ In Japan, nearly two-thirds of all oncologic drug approvals since 2005 have been based on SEPs, with only 17.5% of these studies validated through long-term OS analysis.^4^ Similarly, in the US, 71% of all drug approvals between 2006 and 2022 relied on SEPs.^5^ Pathologic complete response (pCR) is one such SEP, which is defined as the absence of viable tumor within the resected specimen after completion of neoadjuvant therapy.^6^ In 2012, the US Food and Drug Administration (FDA) began supporting the use of pCR as a SEP for long-term outcomes.^6^ Since then, it has been increasingly used in randomized clinical trials (RCTs) comparing neoadjuvant therapies in various disease sites, including breast,^7,8^ rectum,^9^ and other solid tumor sites.^10^

Validation of pCR as a SEP requires testing its correlation with survival at both the patient and trial levels.^11^ Several cancer sites, including breast^12^ and rectal cancer,^13^ have shown a consistent patient-level association with survival. Conversely, in esophagogastric cancers, several trials, including NEO-AEGIS,^14^ TOPGEAR,^15^ and ESOPEC,^16^ have failed to show a patient-level association. Despite the initial encouraging correlation between pCR and survival at the patient level, meta-analyses have failed to show a strong trial-level correlation in many disease sites (correlation coefficient <0.6).^7,9,17^ These discrepancies in the performance of pCR as a SEP across different cancer sites raises concerns that this is a general problem.^18^ Although these meta-analyses were few of the first to question the surrogacy of pCR, they have several limitations that include heterogenous patient populations, outdated treatment regimens, and lack intricate subgroup analyses to draw definitive conclusions.^9^

The use of pCR as a SEP may have important clinical and economic implications by potentially leading to regulatory approval of expensive therapeutics that may not be associated with a true survival benefit. For instance, in rectal cancer, the initial demonstrated patient-level association between pCR and OS in prior studies^19,20,21,22^ has already led to its more widespread use as a SEP in rectal cancer trials using neoadjuvant therapy. There is still no consensus on the trial-level validity of pCR as a SEP in rectal cancer. We performed an updated systematic review and meta-analysis to study the trial-level association between pCR and OS as well as disease-free survival (DFS) in neoadjuvant rectal cancer RCTs.

## Methods

### Search Strategy

This study was conducted in accordance with the Preferred Reporting Items for Systematic Reviews and Meta-Analyses (PRISMA) guidelines.^23^ This study was registered in the International Prospective Register of Systematic Reviews (PROSPERO; CRD42022354557). A systemic literature search of the PubMed, EMBASE, and Cochrane databases was conducted on June 6, 2022, with no date or language exclusions. We used keywords and controlled Medical Subject Heading terms relevant to rectal cancer, neoadjuvant therapy, pCR, and survival. The full search strategy is available in the eAppendix in Supplement 1. The search was repeated before final analysis on January 3, 2024. The final search included RCTs published from database inception to January 3, 2024.

### Study Selection and Inclusion Criteria

Eligible studies were required to satisfy the following criteria: (1) RCT with 2 or more arms, (2) included patients with rectal cancer who underwent neoadjuvant therapy (radiation, chemoradiation, or total neoadjuvant therapy [TNT]) before surgery in both arms, (3) studies with pCR of both arms in the form of absolute numbers or odds ratios (ORs) and 95% CIs or available raw data to calculate them, and (4) studies with survival data presented as hazard ratios (HRs) and 95% CIs or sufficient data to calculate them. Studies with (1) patients who received immunotherapy, (2) patients with metastatic cancer, or (3) results not published at the time of the literature search were excluded. Two independent reviewers (K.S. and J.J.L.) screened the titles and abstracts of the retrieved articles based on the predefined inclusion and exclusion criteria. Full-text articles were then assessed for eligibility. Any discrepancies were resolved through discussion with a third author (Z.V.F.).

### Data Extraction, Quality Assessment, and Outcome Measures

Two independent reviewers (J.J.L. and K.S.) extracted data into a standardized data sheet and recorded the following information: sample size, duration of follow-up, definition of pCR, tumor characteristics, neoadjuvant and adjuvant regimen, pCR rate, and survival outcomes. The primary end point was the correlation between pCR and OS and secondarily with DFS. Adjusted ORs and HRs with their 95% CIs were extracted from included studies.

### Statistical Analysis

Weighted linear regression was used to evaluate the correlation between pCR and OS or DFS. In the regression, the log-transformed ORs and HRs were used because they were assumed to be normally distributed.^24^ The sample size for each study was incorporated as weight in the regression. The β coefficient was used to quantify the correlation. R software, version 4.3.2 (R Foundation for Statistical Computing) was used for statistical analysis.

Assessments of clinical and methodologic heterogeneity were performed. Statistical heterogeneity was assessed with forest plots for visual inspection of CIs for pCR, DFS, and OS, separately. A random-effects meta-analysis was used to account for study heterogeneity. A χ^2^ test for heterogeneity was calculated to evaluate between-study variation using a *P* < .05 threshold. An *I*^2^ statistic was calculated, and the following cut points were used: 25% (low), 50% (moderate), and 75% (high). When heterogeneity was high, an influence plot (Baujat plot) was used to detect the studies that overly contributed to the variation. The funnel plot and Egger test were used to evaluate publication bias. A 2-sided *P* < .05 indicated statistically significant publication bias. Version 2 of the Cochrane Risk of Bias tool for RCTs was used to evaluate risk of bias of all studies.^25^ The overall risk of bias was classified as low, some concerns, or high. A sensitivity analysis was performed excluding studies with a large contribution to the heterogeneity based on the influence plot and those with high risk of bias. The GRADE (Grading of Recommendations Assessment, Development, and Evaluation) tool was used for assessing overall certainty of evidence.^26^

## Results

### Study Characteristics

**Figure 1.**
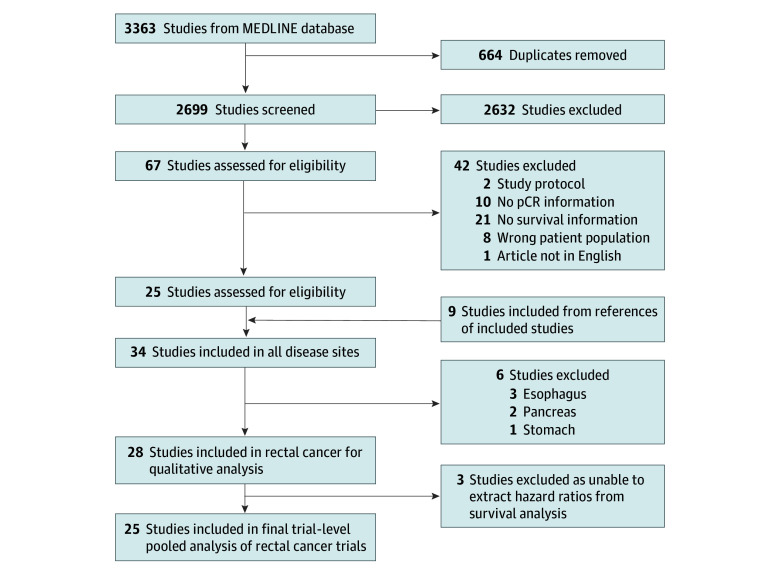
Preferred Reporting Items for Systematic Reviews and Meta-Analyses (PRISMA) Study Flow Diagram pCR indicates pathologic complete response.

Of 3363 identified records, 28 RCTs met the inclusion criteria, contributing to a total of 12 160 patients (Figure 1). Three studies^27,28,29^ did not have pertinent survival data and were excluded from the meta-analysis. The final meta-analysis was performed in 25 RCTs with a total of 11 882 patients (Table).

**Table.** Study Characteristics

Source	Trial name	Phase	Sample size	Included TNM stage	Neoadjuvant arm		Adjuvant therapy	Follow up, median, mo	OR for pCR (95% CI)	HR for OS (95% CI)	HR for DFS (95% CI)
					1	2					
Gerard et al,^30^ 2006	FFCD 9203	2	674	T_3-4_, N_any_, M_0_	RT	CRT (fluorouracil)	Yes	81	3.43 (1.8-6.53)	0.96 (0.85-1.12)	NA
Braendengen et al,^31^ 2008	NA	3	156	T_4_, N_any_, M_0_	RT	CRT (fluorouracil)	Yes	61	0.50 (0.2-1.25)		1.36 (0.88-2.11)
Fernández -Martos et al,^32^ 2010	NA	2	100	T_3-4_, N_any_, M_0_	CRT	TNT (CAPOX following by CRT)	Yes	22	0.97 (0.32-2.91)	1.04 (0.7-1.52)	1.18 (0.46-3.05)
Pach et al,^33^ 2011	NA	2	154	T_3-4_, N_any_, M_0_	Surgery 7 d after RT	Surgery 4-5 wk after RT	Yes	86	12.67 (1.6-100.7)		1.01 (0.4-2.55)
Dewdney et al,^34^ 2012	EXPERT-C	2	90	T_3-4_, N_any_, M_0_	CRT (capecitabine and oxaliplatin)	CRT (capecitabine, oxaliplatin, and cetuximab)	Yes	37 (arm 1), 34 (arm 2)	1.67 (0.37-7.44)	0.27 (0.26-0.99)	
Hofheinz et al,^35^ 2012	NA	3	147	T_3-4_, N_any_, M_0_	CRT (capecitabine)	CRT (fluorouracil)	Yes	52	0.36 (0.11-1.21)	1.28 (0.83-1.54)	
Ngan et al,^36^ 2012	NA	3	315	T_3-4_, N_any_, M_0_	CRT (short course)	CRT (long course)	Yes	71	13.39 (3.1-57.83)	1.12 (0.87-1.29)	1.15 (0.89-1.27)
Mohiuddin et al,^27^ 2013	RTO-0012	2	103	T_3-4_, N_any_, M_0_	CRT (fluorouracil)	CRT (fluorouracil and irinotecan)	Yes	76.8 (arm 2), 84(arm 2)	0.84 (0.35-1.98)		
Appelt et al,^37^ 2014	NA	3	221	T_3-4_, N_any_, M_0_	CRT	CRT and brachytherapy boost	Yes	65	1.01 (0.51-2.01)	0.97 (0.75-1.27)	0.97 (0.74-1.26)
Bosset et al,^38^ 2014	EORTC 22921	2	946	T_3-4_, N_any_, M_0_	RT	CRT (fluorouracil)	Yes	65	2.84 (1.76-4.59)	1.03 (0.91-1.17)	
Aldo et al, 2014	I-CNT-RT	3	588	T_3-4_, N_any_, M_0_	CRT	CRT and adjuvant therapy	Yes	63	1.12 (0.74-1.71)	1.04 (0.88-1.19)	0.97 (0.85-1.15)
Rödel et al,^39^ 2015	CAO/ARO/AIO-04	3	1211	T_3-4_, N_any_, M_0_	CRT (fluorouracil)	CRT (fluorouracil and oxaliplatin)	Yes	50	1.39 (1.02-1.91)	0.96 (0.85-1.13)	0.97 (0.8-0.99)
Allegra et al,^40^ 2015	NSABP R-04	3	1567	T_3-4_, N_any_, M_0_	CRT (fluorouracil)	CRT (capecitabine)	Yes	NA	1.20 (0.93-1.55)	1 (0.86-1.09)	1 (0.87-1.15)
Jung et al,^41^ 2015	NA	2	141	T_3-4_, N_any_, M_0_	CRT (fluorouracil)	CRT (irinotecan and S1)	Yes	43.8	1.75 (0.75-4.07)	2.04 (0.63-6.66)	1.08 (0.56-2.1)
Bujko et al,^42^ 2016	NA	3	425	T_3-4_, N_any_, M_0_	TNT (RT followed by FOLFOX)	CRT (RT + FOLFOX)	Unknown	35	0.82 (0.47-1.43)	0.73 (0.73-1)	0.96 (0.87-1.11)
Latkauskas et al,^43^ 2016	NA	3	140	T_3-4_, N_any_, M_0_	RT	CRT (fluorouracil)	Yes	39.7	2.71 (0.69-10.67)	1.64 (0.89-1.85)	2.20 (0.97-2.26)
Rayan et al,^28^ 2018	NA	2	65	T_3-4_, N_any_, M_0_	CRT (capecitabine and oxaliplatin)	CRT (fluorouracil)	No	40	0.64 (0.21-1.97)	NA	NA
Deng et al,^44^ 2019	FOWARC	3	445	T_3-4_, N_any_, M_0_	CRT (fluorouracil)	CRT (FOLFOX)	Yes	45.2	2.35 (1.3-4.26); 0.44 (0.2-0.97)	1.02 (0.74-1.38); 1.06 (0.76-1.4)	0.84 (0.74-1.13); 0.94 (0.79-1.19)
Fokas et al,^45^ 2019	CAO/ARO/AIO-12	2	306	T_3-4_, N_any_, M_0_	TNT (CT followed by CRT)	TNT (CRT followed by CT)	Yes	43	1.62 (0.93-2.82)	1.1 (0.73-1.51)	0.95 (0.79-1.2)
Schmoll et al,^46^ 2020	PETACC 6	3	1020	T_3-4_, N_any_, M_0_	CRT (capecitabine)	CRT (capecitabine and oxaliplatin)	Yes	68	1.24 (0.86-1.79)	1.12 (0.95-1.25)	1.02 (0.91-1.13)
Francois et al,^47^ 1999; Glehen et al,^48^ 2017	Lyon R90-01	2	201	T2-3, N_any_, M_0_	2 wk between RT and surgery	6-8 wk between RT and surgery	Unknown	35	2.09 (0.81-5.42)	0.95 (0.66-1.36)	0.93 (0.42-2.06)
Azria et al,^49^ 2017	ACCORD 12	3	580	T_3-4_, N_any_, M_0_	CRT (capecitabine)	CRT (capecitabine and oxaliplatin)	Yes	60.2	1.5 (0.96-2.33)	0.82 (0.75-1.1)	1.02 (0.87-1.17)
Wang et al,^29^ 2019	FDRT-002	2	110	T_3-4_, N_any_, M_0_	CRT	CRT + boost	Yes	42	1.83 (0.7-4.8)	NA	NA
Valentini et al,^50^ 2019	INTERACT	3	514	T_2-3_, N_any_, M_0_	CRT (capecitabine)	CRT (capecitabine and oxaliplatin)	Yes	68	0.96 (0.64-1.44)	1.05 (0.87-1.27)	1.01 (0.83-1.22)
Salazar et al,^51^ 2020	NA	2	89	T_3-4_, N_any_, M_0_	CRT (capecitabine)	CRT (capecitabine and bevacizumab)	Yes	NA	1.87 (0.56-6.25)	1.60 (0.78-2.05)	0.68 (0.33-1.39)
Conroy et al,^52^ 2021	PRODIGE 23	3	427	T_3-4_, N_any_, M_0_	CRT (capecitabine)	TNT (FOLFIRINOX and CRT [capecitabine])	Yes	46.5	2.8 (1.69-4.66)	0.65 (0.63-1.02)	0.69 (0.7-0.98)
Bahadoer et al,^53^ 2021	RAPIDO	3	826	T_2-4_, N_any_, M_0_	CRT (capecitabine)	TNT (RT and CAPOX/FOLFOX)	Yes	55.2	2.77 (1.95-3.94)	0.92 (0.82-1.12)	0.75 (0.77-0.97)
Jin et al,^54^ 2022	STELLAR	3	599	T_3-4_, N_any_, M_0_	CRT (CAPOX)	TNT (RT and CAPOX)	Yes	35	0.51 (0.33-0.79)	0.67 (0.67-0.98)	NA

Abbreviations: RT, radiotherapy; CT, chemotherapy; CRT, chemoradiotherapy; NA, not available; S1, tegafur, gimeracil, and oteracil; FOLFOX, folinic acid, fluorouracil, and oxaliplatin; FOLFIRINOX, folinic acid, fluorouracil, irinotecan, and oxaliplatin; CAPOX, capecitabine and oxaliplatin; HR, hazard ratio; OR, odds ratio; pCR, pathologic complete response; OS, overall survival; DFS, disease-free survival; TNT, total neoadjuvant therapy.

### Pathologic Complete Response

All studies reported pCR, which was uniformly defined as yT_0_N_0_ (no residual tumor or lymph node metastasis). The pooled OR of pCR is presented in eFigure 1A in Supplement 1. Bahadoer et al^53^ and Jung et al^41^ contributed to significant heterogeneity (eFigure 2A in Supplement 1). After excluding these studies, the pooled pCR OR was 1.46 (95% CI, 1.11-1.92) (eFigure 3A in Supplement 1).

### pCR vs OS

**Figure 2.**
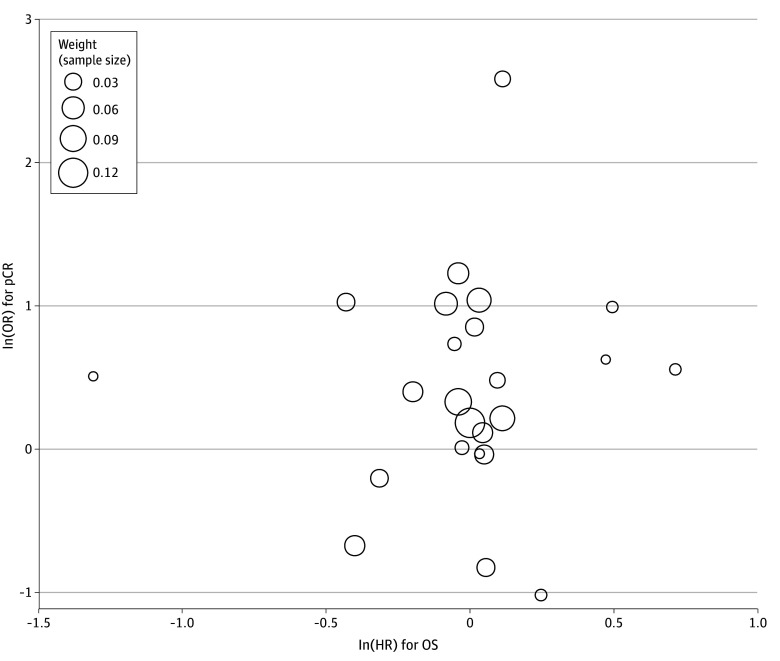
Meta-Regression Comparing Pathologic Complete Response (pCR) and Overall Survival (OS) Meta-regression to evaluate study-level association between treatment effects on pCR and OS. Treatment effects are expressed as odds ratios (ORs) for pCR and log hazard ratios (HRs) for OS. Each circle represents a comparison of the experimental group with the control group, with the size of the circles representing the weight of the comparison, directly proportional to the number of patients in each study.

Twenty-three studies reported OS (eFigure 1B in Supplement 1). After excluding the study by Jung et al^41^ that contributed to significant heterogeneity, the pooled OS HR was 0.98 (95% CI, 0.92-1.05) (eFigure 2B and 3B in Supplement 1). pCR was plotted against OS to study the correlation between the two. On weighted regression analysis, pCR was not correlated with OS (β = 0.37; 95% CI, −0.98 to 1.71; *P* = .57) (Figure 2).

### pCR vs DFS

**Figure 3.**
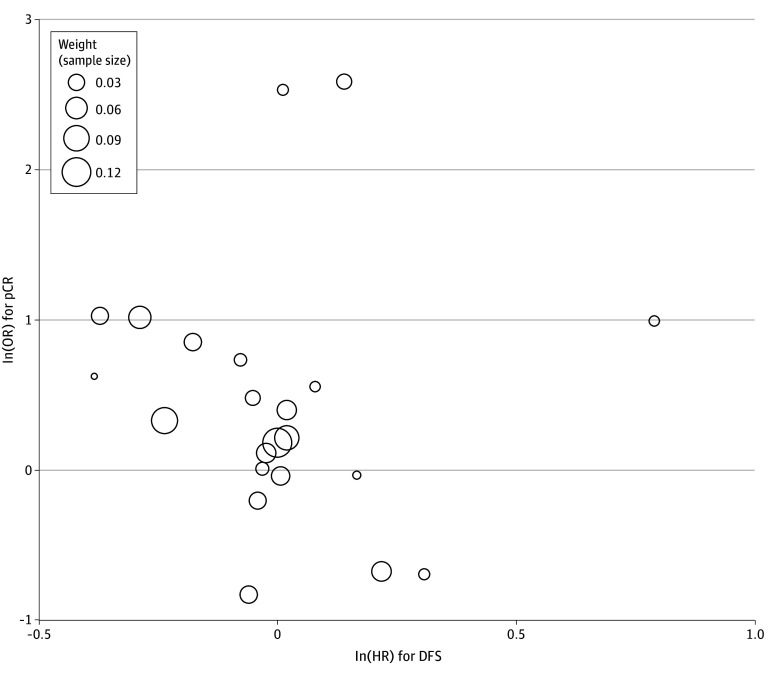
Meta-Regression Comparing Pathological Complete Response (pCR) and Disease-Free Survival (DFS) Meta-regression to evaluate study-level association between treatment effects on pCR and DFS. Treatment effects are expressed as odds ratios (ORs) for pCR and log hazard ratios (HRs) for DFS. Each circle represents a comparison of the experimental group with the control group, with the size of the circles representing the weight of the comparison, directly proportional to the number of patients in each study.

Twenty-one studies reported DFS (eFigure 1C in Supplement 1). After excluding the studies by Braendengen et al^31^ and Jin et al^54^ that contributed to significant heterogeneity, pooled OS HR was 0.94 (95% CI, 0.88-1.01) (eFigures 2C and 3C in Supplement 1). On weighted regression analysis, pCR was not correlated with DFS (β = −0.84; 95% CI, −2.55 to 0.87; *P* = .32) (Figure 3).

### Risk of Bias and Sensitivity Analysis

**Figure 4.**
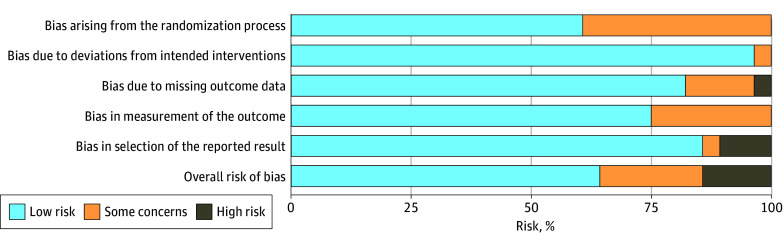
Risk of Bias

The overall risk of bias was low, some concerns, and high in 18, 6, and 4 studies, respectively (Figure 4; eTable 1 in Supplement 1). Among studies with high risk of bias, 2 studies^28,29^ were excluded from the pooled meta-regression due to missing data. Of the remaining 2 studies, the study by Fernández-Martos et al^32^ had a smaller sample size, included patients who did not undergo resection in survival analysis, and had unknown randomization details. The study by Allegra et al^40^ was categorized as high risk due to a discrepancy in sample sizes between pCR and survival analyses and potential inclusion of patients who did not undergo resection in survival analysis.^55^ Sensitivity analysis was performed after excluding these studies with similar results (pCR vs OS: β = 0.40; 95% CI, −1.00 to 1.81; *P* = .55; pCR vs DFS: β = −0.79; 95% CI = −2.63 to 1.04; *P* = .38) (eFigure 4 in Supplement 1). Sensitivity analysis was performed after excluding studies that contributed to significant heterogeneity. pCR was not associated with OS or DFS (eFigure 5 in Supplement 1).

### Subgroup Analysis in Studies Including Only Patients Undergoing Curative Resection in Survival Analysis

Ten studies (40%) included patients who did not undergo curative resection in survival analyses, contributing to slightly different sample sizes when comparing pCR and survival estimates.^30,31,35,39,42,44,46,52,53,56^ This cohort of patients was small, accounting for less than 15% of included patients per study. On subgroup analysis after excluding these studies, pCR was still not correlated with OS or DFS (eFigure 6 in Supplement 1).

### Subgroup Analysis After Excluding Studies That Did Not Use Proportional HRs

Studies were assessed for use of proportional HRs in survival analysis or methods to overcome nonproportionality.^57,58^ Eleven studies (48%) had nonoverlapping survival curves or cited methods to overcome nonproportional HRs when Kaplan-Meier curves overlapped.^30,31,33,34,35,37,46,49,52,54,59,60^ On exclusion, pCR was not correlated with OS or DFS (eFigure 7 in Supplement 1).

### Subgroup Analysis After Excluding Studies That Included Neoadjuvant Radiation Therapy Alone

Although most studies included chemoradiation therapies,^36,51^ 7 studies^30,31,38,43,47,48,49,56^ evaluated neoadjuvant radiation alone in historical cohorts. On subgroup analysis, pCR was not correlated with OS or DFS (eFigure 8 in Supplement 1).

### Publication Bias and Overall Certainty of Evidence

The funnel plot and Egger test did not reveal any asymmetry concerning for publication bias (eFigure 9 in Supplement 1). Including studies with minor discrepancy in sample size between pCR and survival analysis (due to inclusion of patients who did not undergo curative resection in survival analysis) may contribute to imprecision of this meta-analysis. After excluding these studies, overall GRADE certainty of evidence was high for both end points for the remaining 14 studies (eTable 2 in Supplement 1).

## Discussion

In this systematic review and meta-analysis, we comprehensively evaluated the trial-level correlation between pCR and both OS and DFS in rectal cancer RCTs investigating neoadjuvant therapies. No correlation was found between pCR and improved OS or DFS in patients with rectal cancer who underwent neoadjuvant treatment. Results were consistent on sensitivity analyses after excluding studies with high risk of bias. Subgroup analyses were used to examine potential sources of heterogeneity. On excluding historical cohorts with neoadjuvant radiation therapy only and studies including patients who did not undergo resection on survival analysis, pCR was still not associated with DFS and OS.

With increasing clinical studies examining neoadjuvant treatment in rectal cancer, there is increasing emphasis on optimizing efficiency and cost-effectiveness in clinical trials to expedite the approval and implementation of evolving management strategies. One avenue to achieve this is the exploration of surrogate markers for OS, a strategy geared toward expediting trial timelines.^61^ Drawing a parallel from breast cancer research, the FDA stated that pCR can be used as an end point only for accelerated approval and not traditional approval, given that “trial-level relationship between improvement in pCR and improvement in long-term outcome has not been established.”^62,63^ This cautious stance aligns with the findings of 2 large meta-analyses^7,64^ examining the validity of pCR as a surrogate measure for breast cancer, both of which could not confirm PCR as a valid SEP for improved OS.

In rectal cancer research, 3 meta-analyses^50,65,66^ have examined the role of pCR as a factor associated with OS. These analyses collectively underscore the association between pCR and improved OS, but they only focused on patient-level associations, neglecting an exploration of pCR’s surrogacy that requires trial-level association as well. A meta-analysis by Petrelli and colleagues^9^ attempts to bridge this gap by investigating both patient- and trial-level associations. Their conclusion, which contrasts with prior studies,^50,65,66^ asserts that pCR does not serve as a reliable surrogate for OS. However, the meta-analysis used survival probabilities at certain arbitrary time points (3-year DFS and 5-year OS) extracted from the Kaplan-Meier curves, which is a suboptimal statistical estimate for comparison. When HRs used in trials are proportional, comparing them at all possible time points yields a better powered analysis.^67^ Historical cohorts that compared radiation therapy alone in one or both treatment arms were included, where the overall incidence of pCR is low. This study went beyond the aforementioned limitations and benefited from the inclusion of more recent studies^52,53,54^ investigating TNT with pCR rates as high as 15% to 20%. Furthermore, this analysis adopts a more nuanced approach, using relative differences in ORs and HRs of survival curves, which we believe offers a more reflective representation of how survival end points are reported in clinical trials.

Establishing a trial-level association of pCR with survival in addition to patient-level association is critical because these associations do not always translate. This paradox has been discussed in the past and has led to concerns in the medical community regarding adoption of pCR as a surrogate marker in clinical trials.^11,18,45,61,68^ Without convincing trial-level data, the adoption of pCR as a surrogate marker can lead to the approval of therapies that confer toxic effects without improving OS or early abandonment of therapies that may confer long-term benefit. Indeed, studies evaluating trial-level associations are lacking in rectal cancer. There are several possible reasons for the lack of surrogacy of pCR. pCR estimates the effect of therapy on the primary tumor site only and not micrometastases, thereby not effectively estimating which patients may still succumb to distant failure.^69^ Effective neoadjuvant therapies may also not result in a complete response but still have favorable long-term outcomes.^68^ Treatment response in lymph nodes may be a better factor associated with survival than primary tumor response; Pereira and colleagues^70^ performed a retrospective analysis in patients who underwent curative resection after neoadjuvant therapy in patients with gastric cancer. Lymph node response determined based on percentage of fibrosis and residual tumor on examination was associated with DFS. Finally, in many RCTs, patients receive adjuvant therapy, particularly in those not achieving pCR, hence altering the association between pCR and survival.^61,71^

Currently, the paradigm of treating locally advanced rectal cancer has shifted toward TNT after the results of several phase 3 trials during the past decade, which has shown better outcomes compared with conventional chemoradiation therapy. In our meta-analysis, although we included 4 trials that used TNT, pCR was still not correlated with survival. This finding is despite recent trials, such as PRODIGE 23^52^ and RAPIDO,^53^ that found a difference in DFS between arms.^52^ This finding suggests that this survival advantage may not likely be due to a difference in pCR between groups. A subgroup trial-level analysis of TNT trials would be ideal to better answer this question but was not possible due to a limited number of studies that included TNT. In addition to using TNT, there have been several breakthroughs in the management of locally advanced rectal cancer. TNT promotes tumor shrinkage and complete clinical response and facilitates organ preservation (eg, in patients requiring abdominoperineal resection) through the watch and wait strategy. In such patients, pCR may be replaced by a combination of complete clinical response and shorter survival estimates, such as PFS or circulating DNA minimal residual disease, as possible SEPs. Nevertheless, pCR is critical to determine the efficacy of neoadjuvant therapy and feasibility of nonoperative management. Conversely, in patients with higher rectal cancers, the recently published PROSPECT trial showed noninferiority of neoadjuvant chemotherapy compared with chemoradiotherapy.^72^ Further research is required to understand the utility of pCR in this cohort.

### Limitations

This study has certain notable limitations. Our results should be interpreted in the context of the heterogeneity of the studies, which have different neoadjuvant treatments; varying modifications to therapy, including intensified and extended regimens; patient demographics; quality of surgery; trials mandating total mesorectal excision; tumor characteristics; adjuvant treatment^73^; and follow-up. To partially account for this heterogeneity, we performed influence plot analysis and subgroup pooled analyses correlating pCR and survival. A major source of bias is that nearly half of the studies included a small percentage of patients who did not undergo resection in the survival analysis, which may have contributed to bias when correlating pCR with survival outcomes. We performed sensitivity analyses after excluding these studies and still did not find a correlation between pCR and OS or DFS. A subgroup analysis of TNT trials was not feasible due to the insufficient number of included studies, which is more pertinent than historical trials. The receipt of postsurgical therapies may improve outcomes in patients without pCR and may dilute its association with survival. Because most current RCTs include patients who undergo adjuvant therapy, it may not be possible to control for this factor in the community population setting and is a limitation similar to previous meta-analyses.^7,9,17^ When performing survival analyses, it is important to assess whether the HRs are proportional at all time points. Certain studies^30,31,33,34,35,37,46,49,52,54,59,60^ had overlapping Kaplan-Meier curves or it was not possible to assess whether proportionality of HRs was assessed. A subgroup analysis excluding these studies showed similar results. As with previous meta-analyses^7,9,17^ that have investigated the trial-level association of SEPs with long-term outcomes, there is limited spread of treatment effects on weighted regression analysis due to small cohorts of trials analyzed, which may result in a false-negative result.^9^ However, our meta-analysis almost doubled the size of the study by Petrelli et al,^9^ increasing from 13 to 25 trials, with unchanged results. Mediation analysis was not possible due to unavailability of patient-level data (eFigure 10 in Supplement 1).

## Conclusions

Our trial-level analysis did not reveal a correlation between pCR and DFS or OS in rectal cancer RCTs with a high level of evidence. Our study’s findings suggest a recommendation against using pCR as a SEP for neoadjuvant therapies in rectal cancer until conclusive trial-level evidence of its association with long-term outcomes is firmly established.

## Notes

Supplement 1. **eTable 1.** Risk of Bias Analysis of Included Studies Using the ROBINS-II Tool **eTable 2.** GRADE Approach to Ascertain Certainty of Evidence **eFigure 1.** Forest Plot of Pooled Pathological Response Rate, Overall Survival, and Disease-Free Survival **eFigure 2.** Influence Plots for Outcomes **eFigure 3.** Forest Plot of Pooled Pathological Response Rate, Overall Survival, and Disease-Free Survival After Excluding Studies Contributing to Significant Heterogeneity **eFigure 4.** Meta-Regression Comparing Pathological Complete Response and Survival After Excluding High Risk of Bias Studies **eFigure 5.** Meta-Regression Comparing Pathological Complete Response and Survival After Excluding Studies That Had Significant Heterogeneity **eFigure 6.** Meta-Regression Comparing Pathological Complete Response And Survival In Studies Only Including Patients Undergoing Curative Resection on Survival Analysis **eFigure 7.** Meta-Regression Comparing Pathological Complete Response and Survival After Excluding Studies That Did Not Utilize Proportional Hazard Ratios In Survival Analysis **eFigure 8.** Meta-Regression Comparing Pathological Complete Response and Survival After Excluding Studies That Evaluated Neoadjuvant Radiation in One or Both Arms **eFigure 9.** Funnel Plot of Outcome Effect Sizes **eFigure 10.** Mediation Analysis Scheme

Supplement 2.
Data Sharing Statement
