# Supplementary material for: Pathologic Complete Response and Survival in Rectal Cancer: A Systematic Review and Meta-Analysis
Source: JAMA Netw Open. 2025 Jul 16;8(7):e2521197. doi: 10.1001/jamanetworkopen.2025.21197 (PMC12268488; doi:10.1001/jamanetworkopen.2025.21197)
Supplement: Supplement 1. — eTable 1. Risk of Bias Analysis of Included Studies Using the ROBINS-II Tool eTable 2. GRADE Approach to Ascertain Certainty of Evidence eFigure 1. Forest Plot of Pooled Pathological Response Rate, Overall Survival, and Disease-Free Survival eFigure 2. Influence Plots for Outcomes eFigure 3. Forest Plot of Pooled Pathological Response Rate, Overall Survival, and Disease-Free Survival After Excluding Studies Contributing to Significant Heterogeneity eFigure 4. Meta-Regression Comparing Pathological Complete Response and Survival After Excluding High Risk of Bias Studies eFigure 5. Meta-Regression Comparing Pathological Complete Response and Survival After Excluding Studies That Had Significant Heterogeneity eFigure 6. Meta-Regression Comparing Pathological Complete Response And Survival In Studies Only Including Patients Undergoing Curative Resection on Survival Analysis eFigure 7. Meta-Regression Comparing Pathological Complete Response and Survival After Excluding Studies That Did Not Utilize Proportional Hazard Ratios In Survival Analysis eFigure 8. Meta-Regression Comparing Pathological Complete Response and Survival After Excluding Studies That Evaluated Neoadjuvant Radiation in One or Both Arms eFigure 9. Funnel Plot of Outcome Effect Sizes eFigure 10. Mediation Analysis Scheme [file jamanetwopen-e2521197-s001.pdf]

Supplemental Online Content

Sugumar K, Lie JJ, Stucky C-C, et al. Pathologic complete response and survival in rectal cancer: a systematic review and meta-analysis. *JAMA Netw Open*. 2025;8(7):e2521197. doi:10.1001/jamanetworkopen.2025.21197

eTable 1. Risk of Bias Analysis of Included Studies Using the ROBINS-II Tool
eTable 2. GRADE Approach to Ascertain Certainty of Evidence
eFigure 1. Forest Plot of Pooled Pathological Response Rate, Overall Survival, and Disease-Free Survival
eFigure 2. Influence Plots for Outcomes
eFigure 3. Forest Plot of Pooled Pathological Response Rate, Overall Survival, and Disease-Free Survival After Excluding Studies Contributing to Significant Heterogeneity
eFigure 4. Meta-Regression Comparing Pathological Complete Response and Survival After Excluding High Risk of Bias Studies
eFigure 5. Meta-Regression Comparing Pathological Complete Response and Survival After Excluding Studies That Had Significant Heterogeneity
eFigure 6. Meta-Regression Comparing Pathological Complete Response And Survival In Studies Only Including Patients Undergoing Curative Resection on Survival Analysis
eFigure 7. Meta-Regression Comparing Pathological Complete Response and Survival After Excluding Studies That Did Not Utilize Proportional Hazard Ratios In Survival Analysis
eFigure 8. Meta-Regression Comparing Pathological Complete Response and Survival After Excluding Studies That Evaluated Neoadjuvant Radiation in One or Both Arms
eFigure 9. Funnel Plot of Outcome Effect Sizes
eFigure 10. Mediation Analysis Scheme

This supplemental material has been provided by the authors to give readers additional information about their work.

© 2025 Sugumar K et al. *JAMA Network Open.*

eTable 1. Risk of Bias Analysis of Included Studies Using the ROBINS-II Tool

Items	Bias from randomization process	Bias due to deviation from intended interventions	Bias due to missing outcome data	Bias in measurement of outcomes	Bias in selection of reported result	**Overall**
Gerard	Some concerns	Low	Low	Low	Low	Low
Braendengen	Some concerns	Low	Low	Low	Low	Low
Fernandez-Martos	Some concerns	Low	Some concerns	Some concerns	Low	High
Pach	Low	Low	Some concerns	Some concerns	Low	Some concerns
Dewdney	Some concerns	Low	Low	Low	Low	Low
Hofheinz	Low	Low	Low	Low	Low	Low
Ngan	Some concerns	Low	Low	Low	Low	Low
Mohiuddin	Some concerns	Low	Low	Low	Low	Low
Appelt	Low	Low	Low	Low	Low	Low
Bosset	Low	Low	Low	Low	Low	Low
Aldo	Low	Low	Low	Low	Low	Low
Rödel	Low	Low	Low	Some concerns	Low	Some concerns
Allegra	Low	Low	Some concerns	Some concerns	High	High
Jung	Some concerns	Low	Low	Low	Low	Low
Bujko	Some concerns	Low	Low	Some concerns	Low	Some concerns
Latkauskas	Some concerns	Low	Low	Some concerns	Low	Some concerns
Rayan	Some concerns	Some concerns	High	Some concerns	High	High
Deng	Low	Low	Low	Low	Low	Low
Fokas	Low	Low	Low	Low	Low	Low
Schmoll	Low	Low	Some concerns	Low	Low	Some concerns
Francoid, Glehen	Low	Low	Low	Low	Low	Low
Azria	Low	Low	Low	Low	Low	Low
Wang	Some concerns	Low	Low	Low	High	High
Valentini	Low	Low	Low	Low	Low	Low
Salazar	Low	Low	Low	Low	Low	Low
Conroy	Low	Low	Low	Low	Some concerns	Some concerns
Bahadoer	Low	Low	Low	Low	Low	Low
Jin	Low	Low	Low	Low	Low	Low

Risk of bias analysis as per Cochrane Risk of Bias Tool, Version 2.

© 2025 Sugumar K et al. *JAMA Network Open.*

eTable 2. GRADE Approach to Ascertain Certainty of Evidence

GRADE Certainty assessment						
**Participants (studies)**	**Risk of bias**	**Inconsistency**	**Indirectness**	**Imprecision**	**Publication bias**	**Overall certainty of evidence**
pCR vs. OS (5451 patients, 14 studies)	not serious	not serious	not serious	not serious	not serious	⊕⊕⊕⊕ HIGH
pCR vs. DFS (5515 patients, 14 studies	not serious	not serious	not serious	not serious	not serious	⊕⊕⊕⊕ HIGH

GRADE: Grading of recommendations Assessment, Development, and Evaluation; pCR: pathological complete response; OS: overall survival; DFS: disease free survival.
*Studies including patients who did not undergo surgery in survival analysis led to discrepancy in sample size between pCR and survival analysis, leading to possible imprecision. These studies were excluded for final GRADE certainty of evidence analysis.

© 2025 Sugumar K et al. *JAMA Network Open.*

eFigure 1. Forest Plot of Pooled Pathological Response Rate, Overall Survival, and Disease-Free Survival

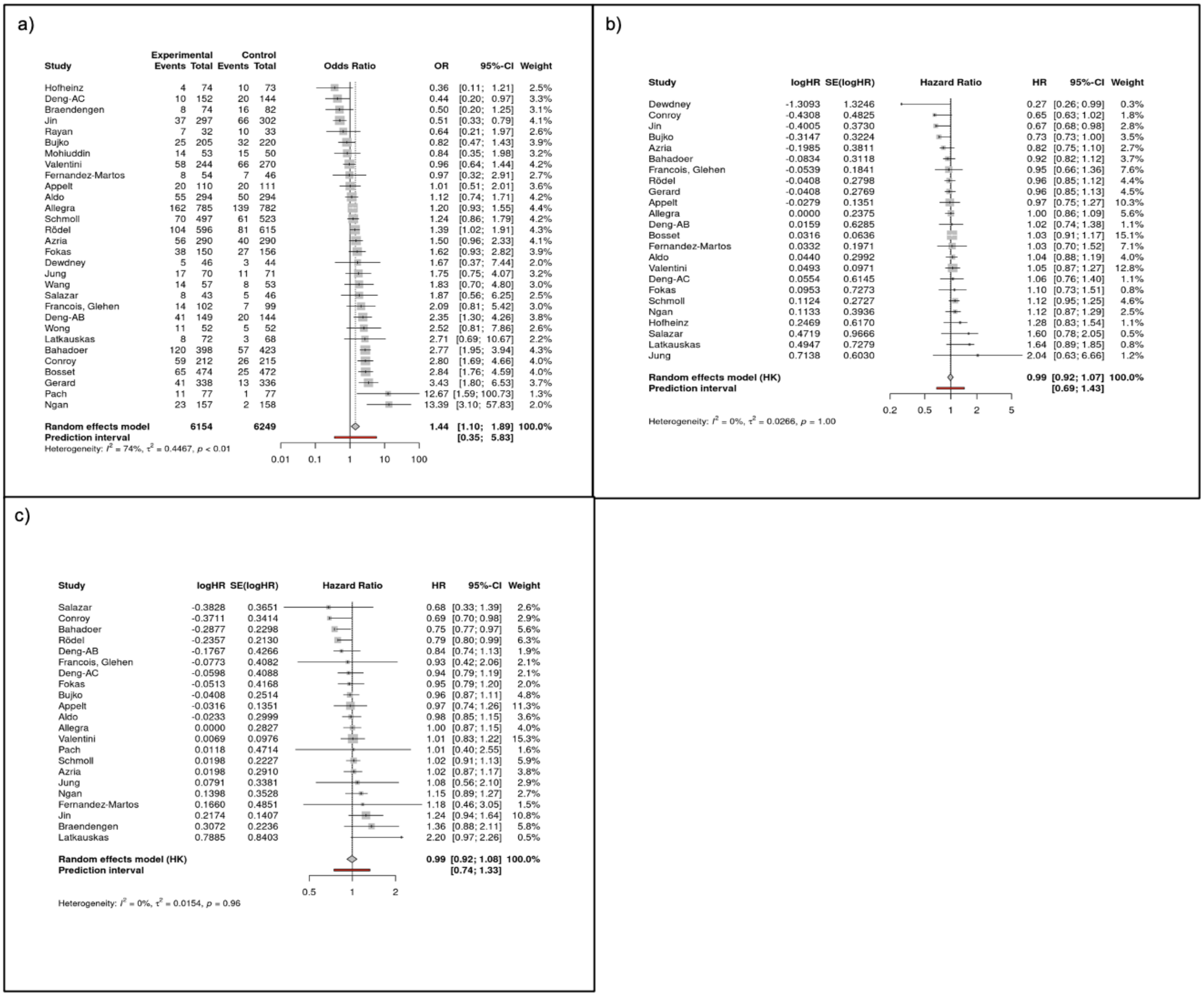

Pooled a) odds ratio for pathological complete response; b) hazard ratio for overall survival and; c) disease-free survival
OR: odds ratio; HR: hazard ratio; SE: standard error; CI: confidence interval

© 2025 Sugumar K et al. *JAMA Network Open.*

eFigure 2. Influence Plots for Outcomes

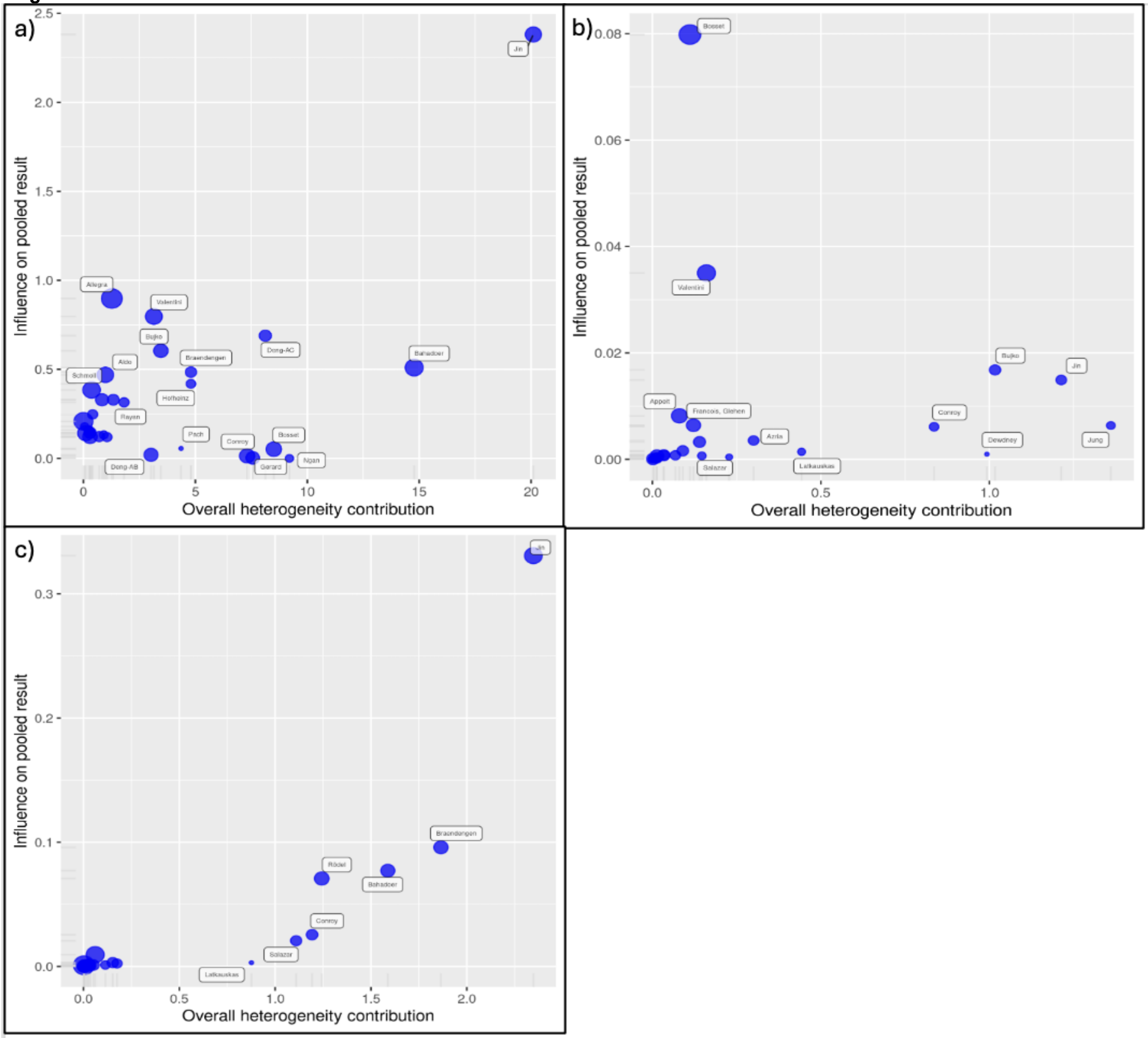

a) pathological complete response; b) overall survival; c) disease-free survival

© 2025 Sugumar K et al. *JAMA Network Open.*

eFigure 3. Forest Plot of Pooled Pathological Response Rate, Overall Survival, and Disease-Free Survival After Excluding Studies Contributing to Significant Heterogeneity

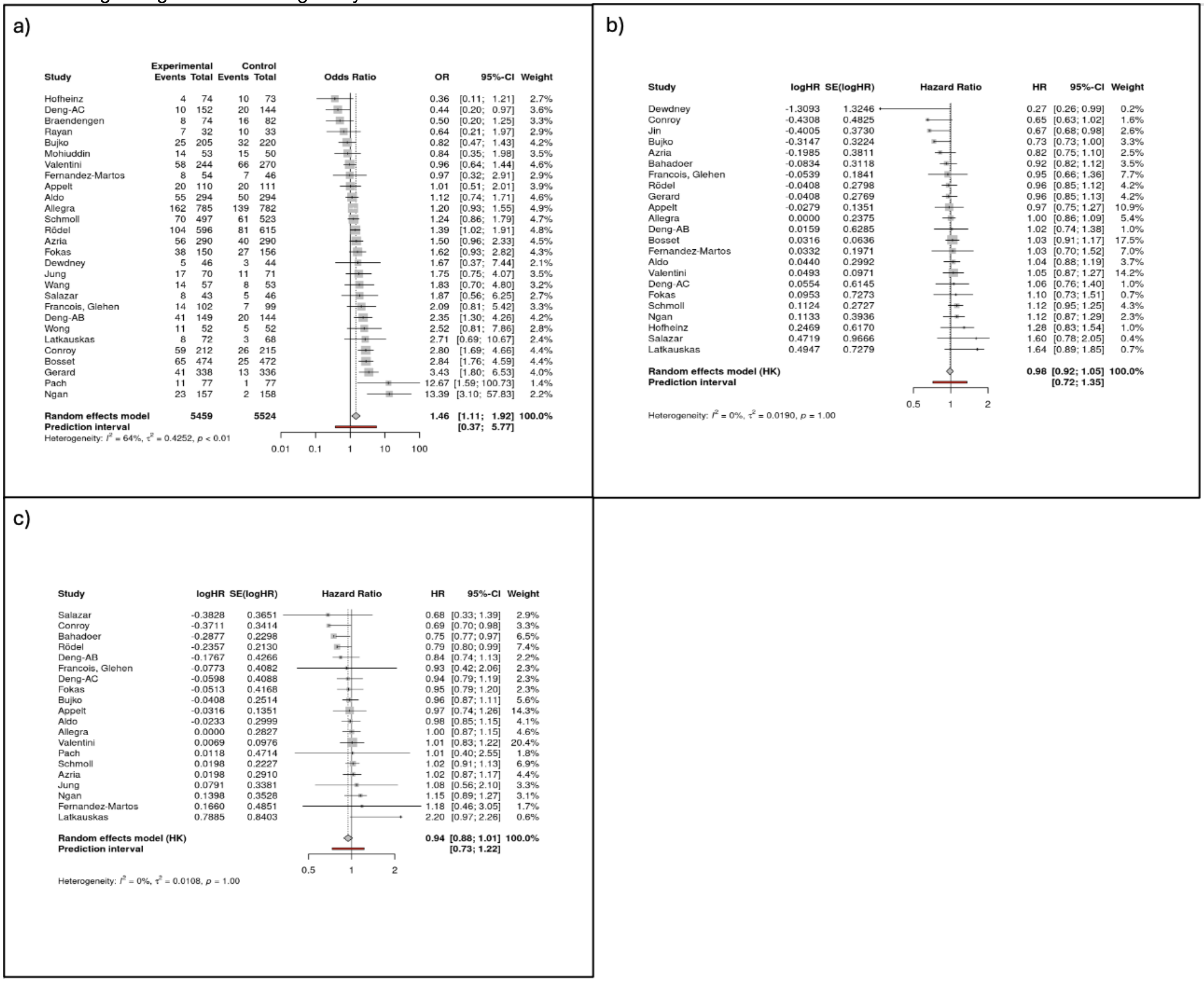

a) pathological complete response; b) overall survival; c) disease-free survival
OR: odds ratio; HR: hazard ratio; SE: standard error; CI: confidence interval

© 2025 Sugumar K et al. *JAMA Network Open.*

eFigure 4. Meta-Regression Comparing Pathological Complete Response and Survival After Excluding High Risk of Bias Studies

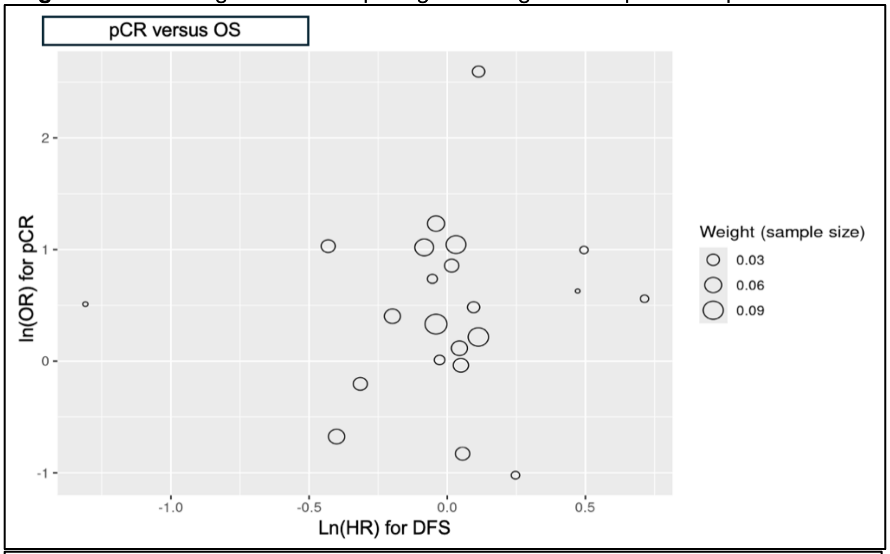

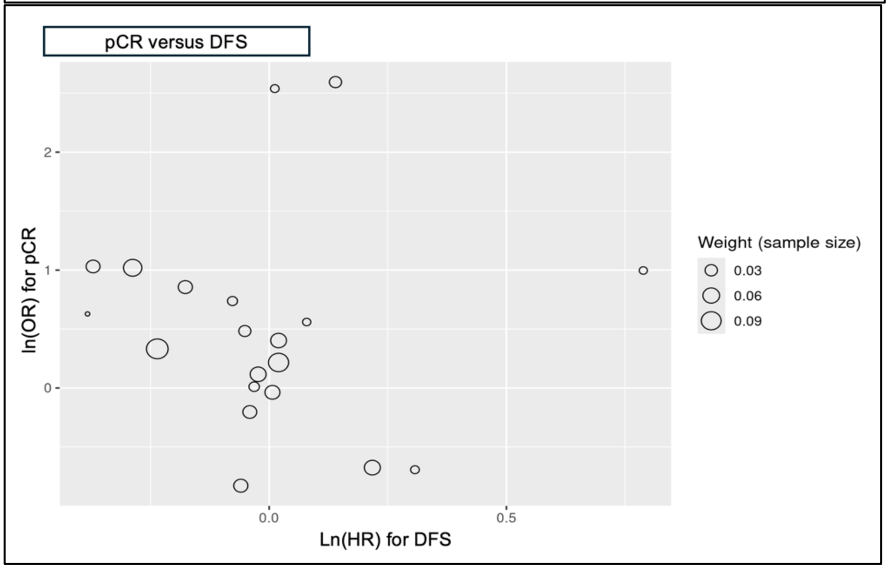

pCR: pathological complete response; OS: overall survival; DFS: disease-free survival; OR: odds ratio; HR: hazards ratio.

© 2025 Sugumar K et al. *JAMA Network Open.*

eFigure 5. Meta-Regression Comparing Pathological Complete Response and Survival After Excluding Studies That Had Significant Heterogeneity

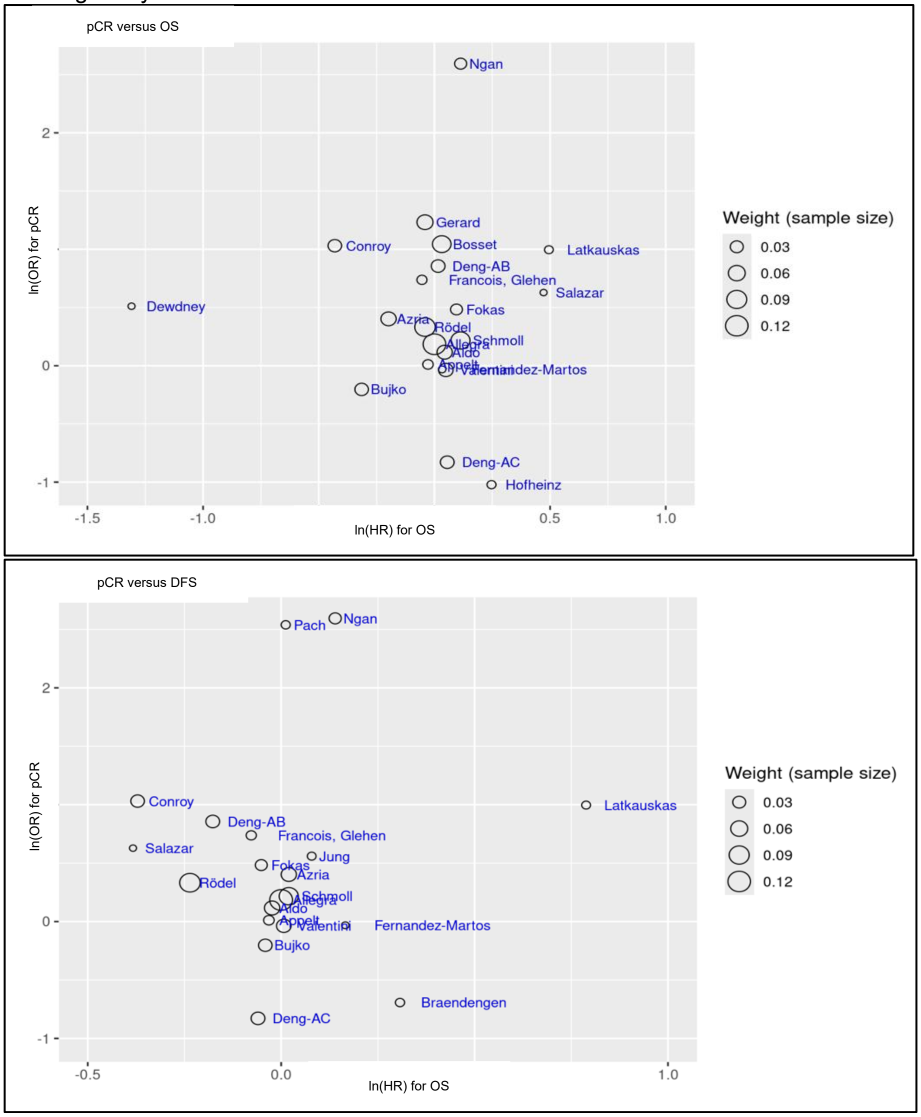

pCR: pathological complete response; OS: overall survival; DFS: disease-free survival; OR: odds ratio; HR: hazards ratio.

a) PCR versus OS, b) PCR versus DFS

© 2025 Sugumar K et al. *JAMA Network Open.*

eFigure 6. Meta-Regression Comparing Pathological Complete Response And Survival In Studies Only Including Patients Undergoing Curative Resection on Survival Analysis

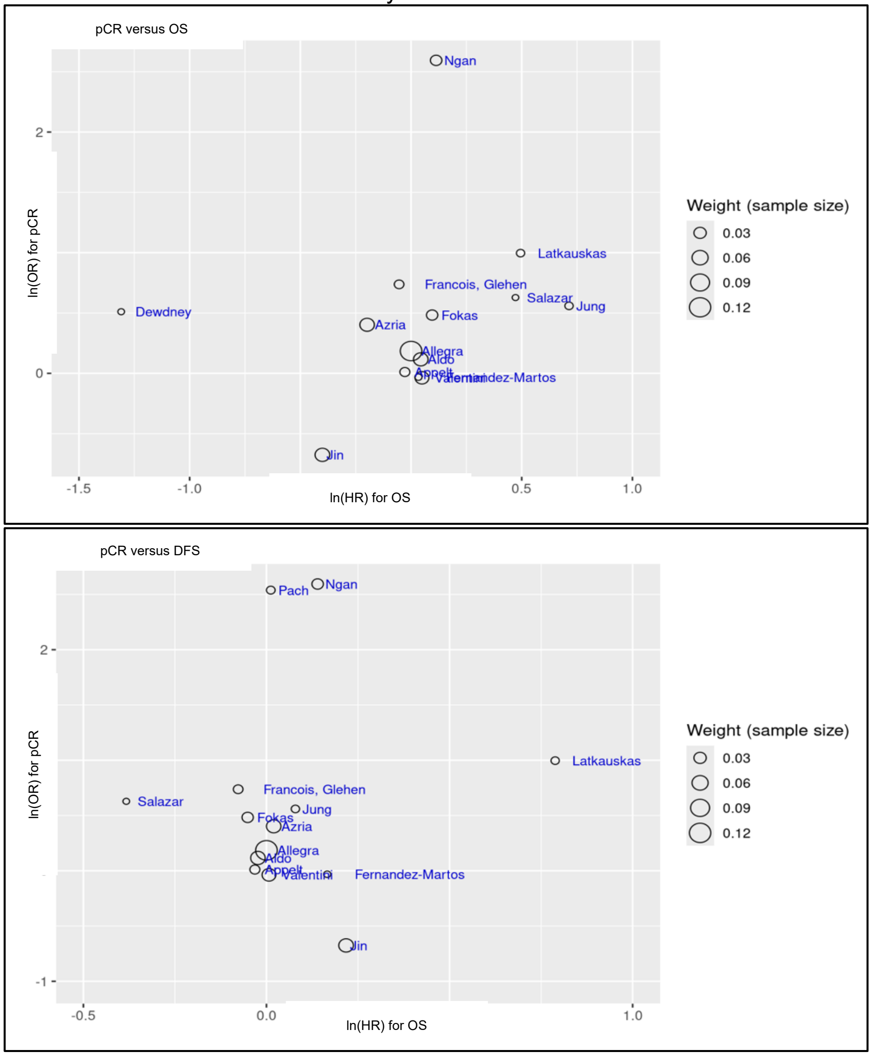

pCR: pathological complete response; OS: overall survival; DFS: disease-free survival; OR: odds ratio; HR: hazards ratio.

© 2025 Sugumar K et al. *JAMA Network Open.*

eFigure 7. Meta-Regression Comparing Pathological Complete Response and Survival After Excluding Studies That Did Not Utilize Proportional Hazard Ratios In Survival Analysis

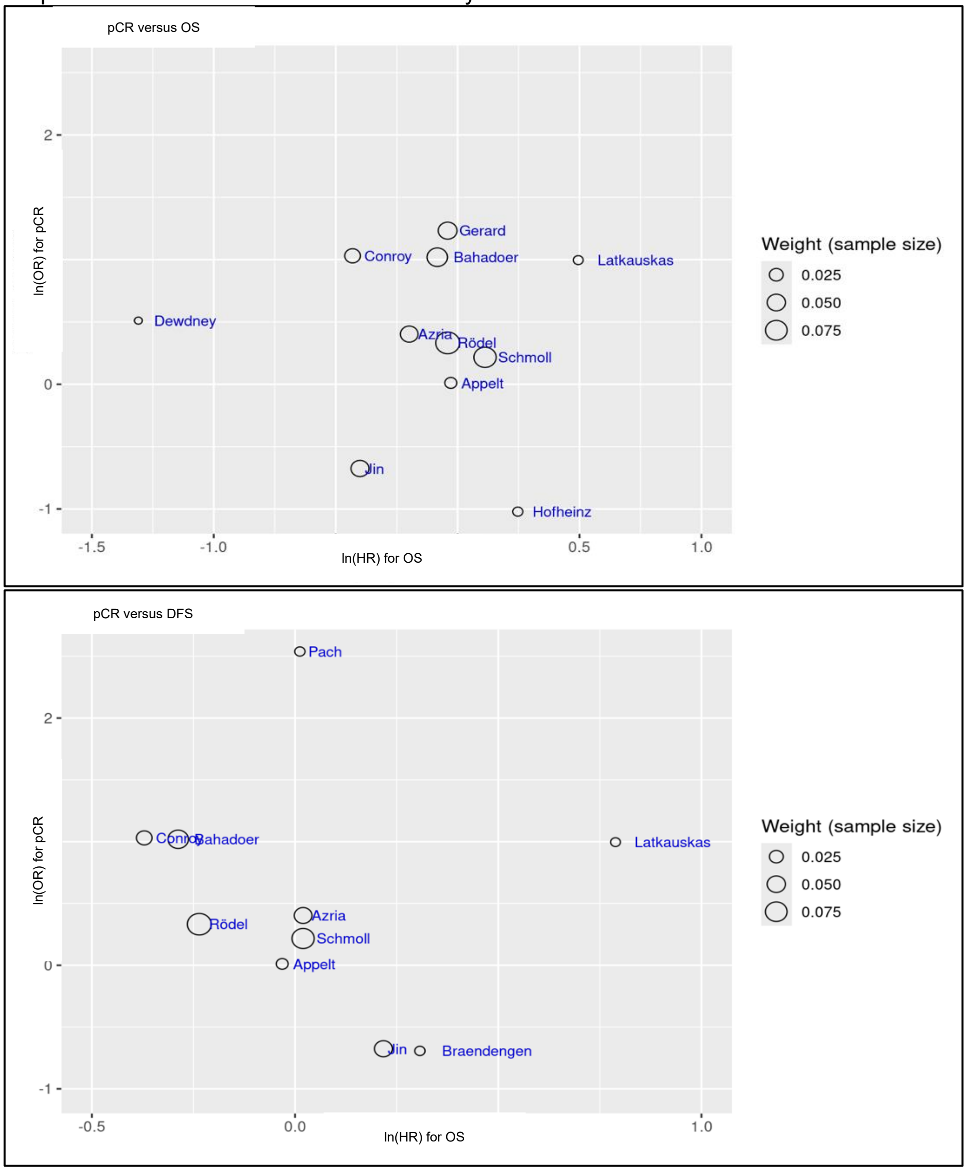

pCR: pathological complete response; OS: overall survival; DFS: disease-free survival; OR: odds ratio; HR: hazards ratio.

© 2025 Sugumar K et al. *JAMA Network Open.*

eFigure 8. Meta-Regression Comparing Pathological Complete Response and Survival After Excluding Studies That Evaluated Neoadjuvant Radiation in One or Both Arms

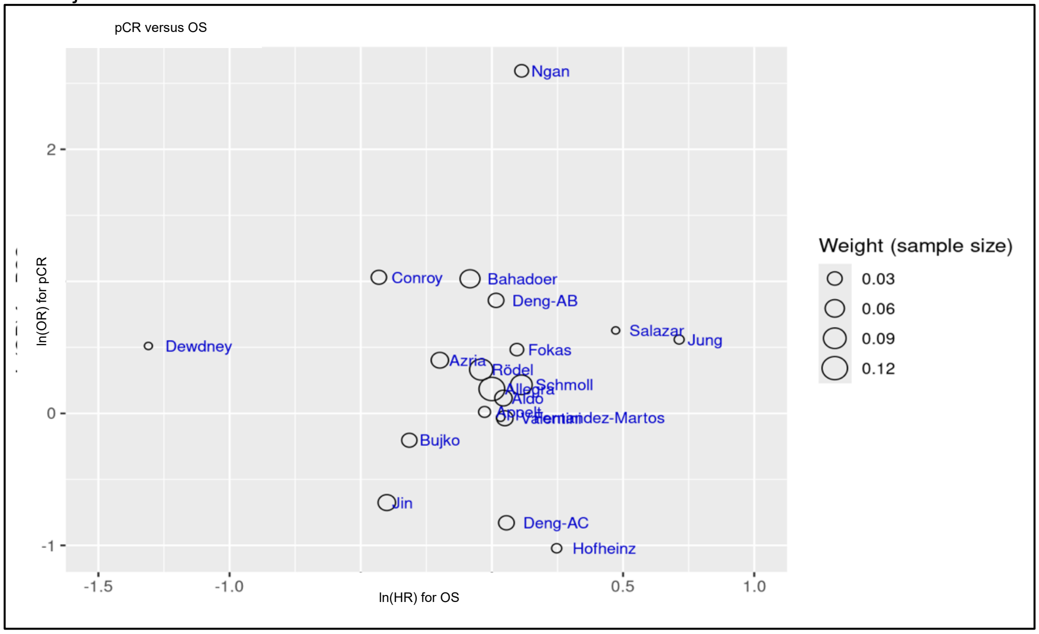

© 2025 Sugumar K et al. *JAMA Network Open.*

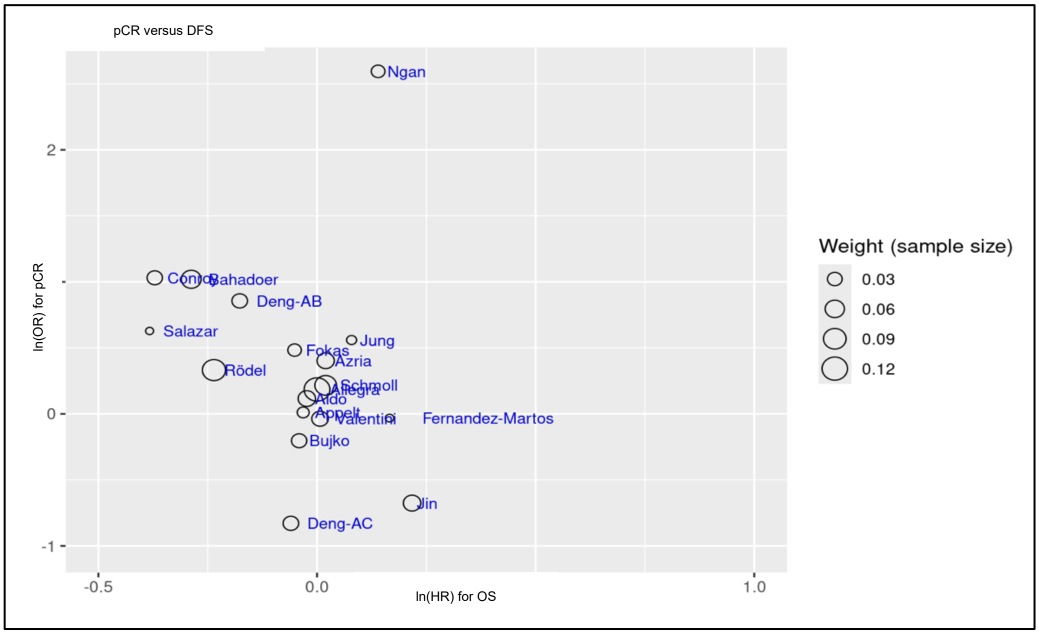

pCR: pathological complete response; OS: overall survival; DFS: disease-free survival; OR: odds ratio; HR: hazards ratio.

eFigure 9. Funnel Plot of Outcome Effect Sizes

© 2025 Sugumar K et al. *JAMA Network Open.*

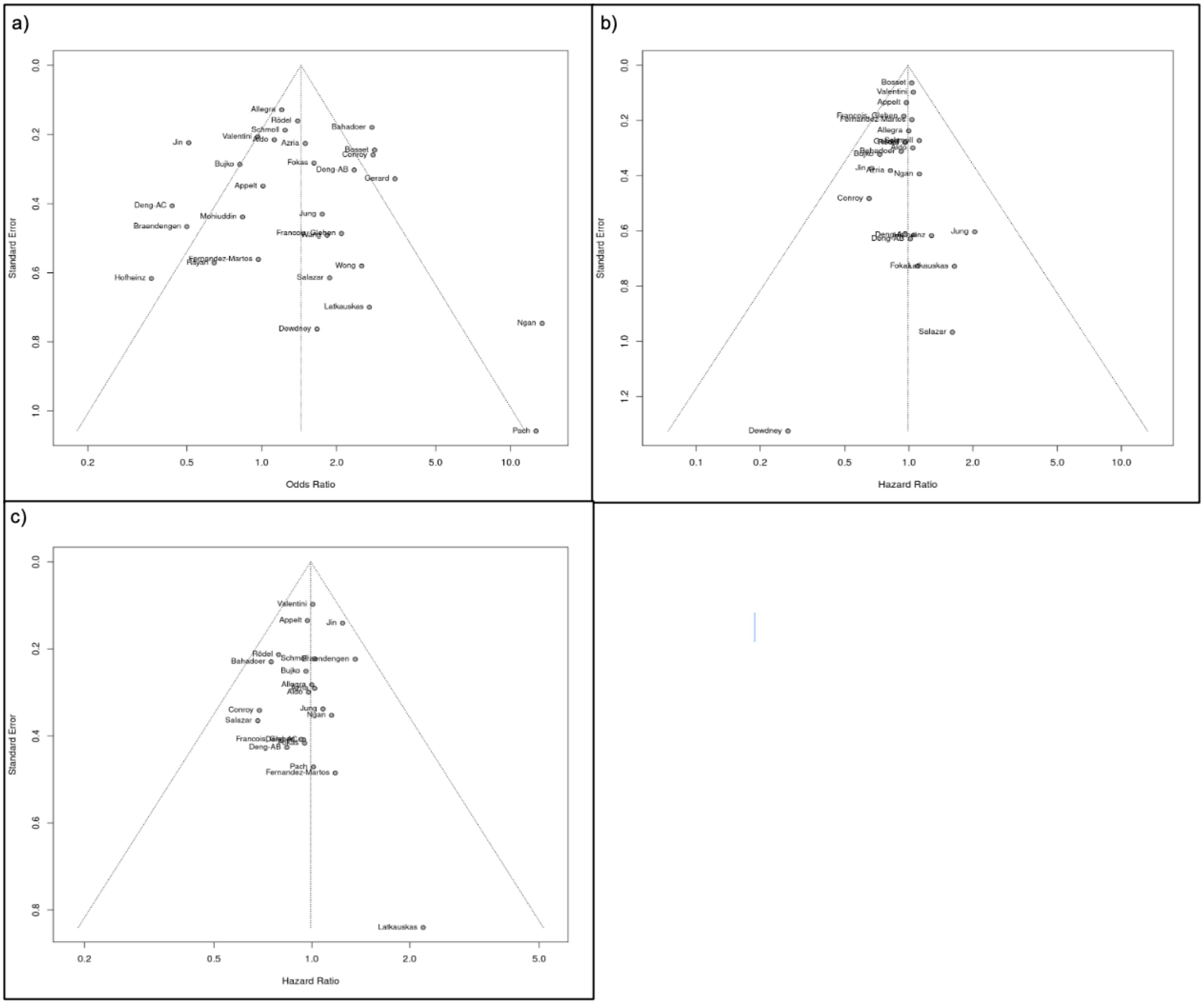

a) pathological complete response; b) overall survival; c) disease-free survival

© 2025 Sugumar K et al. *JAMA Network Open.*

eFigure 10. Mediation Analysis Scheme

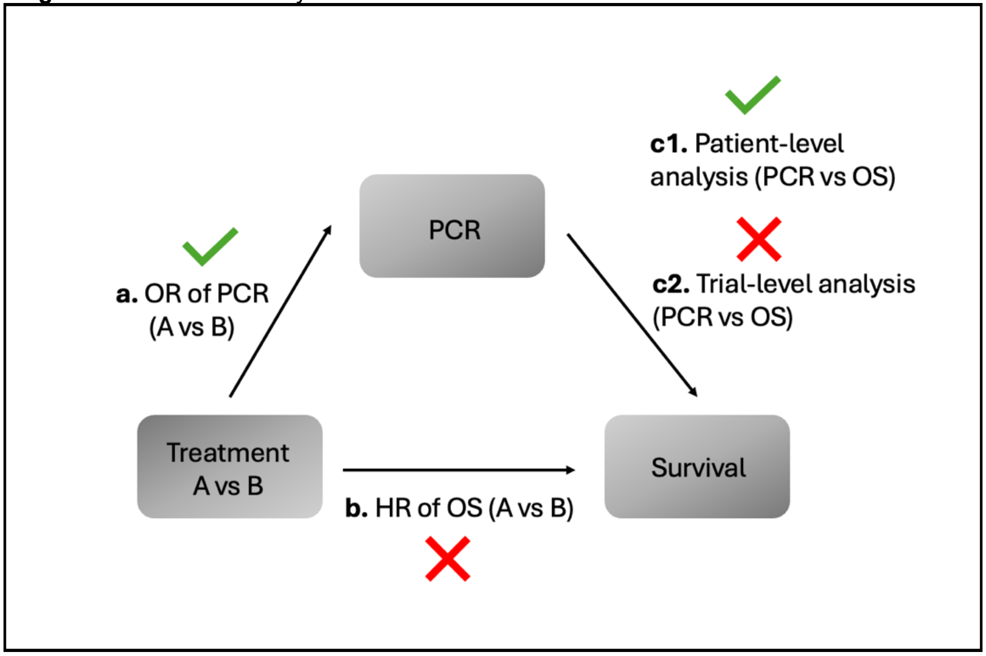

To establish ideal surrogacy comparing treatment groups A & B, we need to show that the relationship between treatment and survival is possible only through the surrogate (pathway a + b; treatment A/B vs pCR [pathological complete response], PCR vs OS [overall survival]); i.e., pCR captures the entire treatment effect of the outcome (survival), and not through pathway b (OS between treatment groups A & B). In our meta-analysis, due to non-availability of patient-level data, it is not possible to perform mediation analysis comparing this.

© 2025 Sugumar K et al. *JAMA Network Open.*
