# Supplementary material for: Pathologic Complete Response and Survival in Rectal Cancer: A Systematic Review and Meta-Analysis
Source: JAMA Netw Open. 2025 Jul 16;8(7):e2521197. doi: 10.1001/jamanetworkopen.2025.21197 (PMC12268488; doi:10.1001/jamanetworkopen.2025.21197)
Supplement: Supplement 2. — Data Sharing Statement [file jamanetwopen-e2521197-s002.pdf]

Data Sharing Statement

Sugumar. Pathologic Complete Response and Survival in Rectal Cancer. *JAMA Netw Open*. Published July 16, 2025. doi:10.1001/jamanetworkopen.2025.21197

Data
Data available: No

Additional Information
Explanation for why data not available: This is a meta-analysis, there is no patient data, rather, they are published aggregate data.
